# Prevalence and clinical characteristics of Sarcopenia in older adult patients with stable chronic obstructive pulmonary disease: a cross-sectional and follow-up study

**DOI:** 10.1186/s12890-024-03034-5

**Published:** 2024-05-02

**Authors:** Sang Hun Kim, Cho Hui Hong, Myung-Jun Shin, Ki Uk Kim, Tae Sung Park, Jun Yong Park, Yong Beom Shin

**Affiliations:** 1 Department of Rehabilitation Medicine, Biomedical Research Institute, Pusan National University Hospital and Pusan National University School of Medicine, Busan, Republic of Korea; 2 Biomedical Research Institute, Pusan National University Hospital, Busan, Republic of Korea; 3 Department of Physical Therapy, Graduate School, Kyungsung University, Busan, Republic of Korea; 4 Department of Internal Medicine, Pusan National University School of Medicine, Busan, Republic of Korea; 5 Department of Rehabilitation Medicine, Biomedical Research Institute, Pusan National University Hospital, Busan, Republic of Korea

**Keywords:** Sarcopenia, Chronic obstructive Pulmonary Disease, Maximal respiratory pressures, Grip Strength

## Abstract

**Background:**

The relationship between sarcopenia and chronic obstructive pulmonary disease (COPD) has been increasingly reported, and there is some overlap regarding their clinical features and pulmonary rehabilitation (PR) strategies. No Korean study has reported the actual prevalence of sarcopenia in patients with stable COPD who are recommended for pulmonary rehabilitation. This study evaluated the prevalence and clinical features of sarcopenia in older adult outpatients with stable COPD and the changes after 6 months.

**Methods:**

In this cross-sectional and 6-month follow-up study, we recruited 63 males aged ≥ 65 diagnosed with stable COPD. Sarcopenia was diagnosed using the AWGS 2019 criteria, which included hand grip strength testing, bioelectrical impedance analysis, Short Physical Performance Battery administration, and Strength, Assistance with walking, Rising from a chair, Climbing stairs, and Falling screening tool administration. A 6-minute walk test (6 MWT) was conducted, forced vital capacity (FVC), forced expiratory volume in 1 s (FEV_1_), maximal inspiratory and expiratory pressures (MIP and MEP, respectively) and peak expiratory flow (PEF) were assessed, and patient-reported questionnaires were administered.

**Results:**

At baseline, 14 (22%) patients were diagnosed with possible sarcopenia, and eight (12.6%) were diagnosed with sarcopenia. There were significant differences in the age; body mass index; Body mass index, airflow Obstruction, Dyspnea, and Exercise index; modified Medical Research Council dyspnea scores; and International Physical Activity Questionnaire scores between the normal and sarcopenia groups. Whole-body phase angle, MIP, MEP, PEF, and 6-minute walk distance (6 MWD) also showed significant differences. Over 6 months, the proportion of patients with a reduced FEV_1_ increased; however, the proportion of patients with sarcopenia did not increase.

**Conclusion:**

A relatively low prevalence of sarcopenia was observed in older adult outpatients with stable COPD. No significant change in the prevalence of sarcopenia was found during the 6-month follow-up period.

**Trial registration:**

The study was registered with the Clinical Research Information Service (KCT0006720). Registration date: 30/07/2021.

**Supplementary Information:**

The online version contains supplementary material available at 10.1186/s12890-024-03034-5.

## Background

Chronic obstructive pulmonary disease (COPD) is characterized by airflow limitation and persistent respiratory symptoms caused by increased airway resistance and damage to the lung parenchyma due to exposure to noxious particles or gases [1]. The social burden caused by COPD is gradually increasing, considering an increasingly aging population [2]. Aging is an independent risk factor for COPD, with a prevalence rate of 14% in adults aged ≥ 65 compared with that of 9.9% in younger people [3]. The prevalence of COPD in Korea has been reported to be 12.9%, and approximately one-third of the population aged > 70 meets the diagnostic criteria for COPD [4].

In 2010, the European Working Group on Sarcopenia in Older People (EWGSOP) proposed an algorithm for diagnosing sarcopenia, which was updated to EWGSOP2 in 2018 [5, 6]. The Asian Working Group for Sarcopenia (AWGS) proposed a revised algorithm for diagnosing sarcopenia in 2019 considering racial differences [7]. The prevalence rates of sarcopenia in Koreans aged ≥ 70, according to the AWGS 2019, were 21.3% in males and 13.8% in females [8]. Sarcopenia is known to cause a decrease in physical activity due to age-related loss of skeletal muscle, resulting in poor health and increased mortality [9]. Sarcopenia is associated with chronic diseases, such as chronic kidney disease and COPD [10, 11]. The prevalence of sarcopenia in patients with COPD was reported to be 25% in Korea [12]. A relationship between sarcopenia and COPD has been increasingly reported, and there is some overlap in clinical features and pulmonary rehabilitation (PR) strategies [13]. However, no Korean study has yet been conducted on the actual prevalence of sarcopenia according to the AWGS 2019 criteria in patients with stable COPD who are recommended for PR. This study evaluated the prevalence and clinical features of sarcopenia according to the AWGS 2019 criteria in older adult outpatients with stable COPD and the changes after 6 months.

## Materials and methods

### Study design and sample size

We performed a single-center cross-sectional study and follow-up evaluation after 6 months. This study was conducted at the regional respiratory center of the Pusan National University Hospital, Korea. This study was conducted according to the recommendations of the Strengthening the Reporting of Observation Studies in Epidemiology statement for observational studies [14]. Based on the results of a previous study [12], the sample size was calculated using G power 3.1. Based on a two-tailed α of 0.05 and power of 0.9, we expected an effect size of 0.91, and a minimum of 63 patients were required considering a 20% rate of loss to follow-up.

### Study population

This study included male patients diagnosed with COPD who visited our regional respiratory center between August 2021 and November 2022. COPD was diagnosed in accordance with the Global Initiative for Chronic Obstructive Lung Disease (GOLD) guidelines. The inclusion criteria were: age ≥ 65, male sex, diagnosis of stable COPD (modified Medical Research Council [mMRC] dyspnea score ≥ 2 or COPD assessment test (CAT) score ≥ 10). The exclusion criteria were: diagnosis of acute respiratory diseases or lung cancer; limited physical activity due to any physical or medical problems; contraindications for bioelectrical impedance analysis (BIA), such as a pacemaker or implantable cardioverter defibrillator; and a history of surgery within the last 3 months. A medical record review was performed for age, sex, smoking history, underlying disease, medication history, GOLD stage, and the number of hospitalizations due to acute exacerbation of COPD in the past year. Written informed consent was obtained from all the participants. This study was approved by the Institutional Review Board (IRB) of the Pusan National University Hospital (IRB number: 2107-012-104).

### Clinical outcomes

Sarcopenia was diagnosed using the AWGS 2019 criteria, which included hand grip strength (HGS) testing, BIA, Short Physical Performance Battery (SPPB) administration, and Strength, Assistance with walking, Rising from a chair, Climbing stairs, and Falling (SARC-F) questionnaire administration. According to AWGS 2019 guidelines, sarcopenia is defined as possible sarcopenia when the HGS is < 28 kg for males and < 18 kg for females in patients with SARC-F scores ≥ 4. The SARC-F is a self-reported questionnaire with five questions, each scored between 0 and 2. The higher the score, the lower the physical function has been reported [15, 16]. In this study, grip strength was measured using a handheld dynamometer (Jamar Plus+; Sammons Preston, Rolyon, Bolingbrook, IL, USA) via a standardized method with elbow flexion at 90° in a sitting position, three times on each side, and the maximum value recorded [17]. In the AWGS 2019 algorithm, physical performance, is also evaluated using the SPPB when patients are diagnosed with possible sarcopenia. The SPPB consists of balance assessment, gait speed measurement, and a 5-time sit-to-stand (5TSTS) test. The balance test was scored based on whether it was possible to maintain a side-by-side stand, semi-tandem stand, or tandem stand for 10 s. Gait speed was calculated by walking 6 m at the usual speed and calculating the speed of walking in the middle 4 m. In the 5TSTS test, the participants sat down and stood up from the chair as quickly as possible five times with their arms folded toward their chest, ending in a standing position, and the time required was measured [18]. All scores were summed, and an SPPB score ≤ 9 was considered to indicate low physical performance. Skeletal muscle mass (SMM) was measured using BIA. BIA was performed by connecting the electrodes to the wrist and ankle after resting for 3 min in the supine position using a segmental multi-frequency BIA system (S10, InBody Co., Ltd, Seoul, South Korea). The SMM index (SMI) was calculated by dividing the SMM (kg) by height squared (m^2^). Low muscle mass was defined as an SMI < 7.0 kg/m^2^ in males and < 5.7 kg/m^2^ in females. According to the AWGS 2019 algorithm, sarcopenia was defined as low muscle mass, HGS, or physical performance; in contrast, severe sarcopenia was defined when all three parameters were low.

A 6-minute walk test (6 MWT) was conducted, forced vital capacity (FVC), forced expiratory volume in 1 s (FEV_1_), maximal inspiratory and expiratory pressures (MIP and MEP) and peak expiratory flow (PEF) were assessed, and patient-reported questionnaires, such as the International Physical Activity Questionnaire (IPAQ), Lung Information Needs Questionnaire (LINQ), and London Chest Activity of Daily Living (LCADL) were administered. Participants underwent a 6 MWT on a 30-m straight corridor with a distance marked according to standardized guidelines [19]. FVC, FEV_1_, MIP and MEP were evaluated using a standardized method with a desktop spirometer (Pony FX, Cosmed, Rome, Italy) [20]. Three measurements were obtained, with participants in a sitting position, using a flange-type mouthpiece, and the maximal measurement was recorded. The PEF was measured using a peak flow meter (Peak Flow Meter; Clement Clarke, UK). The maximum value obtained from three trials was recorded [21]. One physical therapist performed all evaluations, which were repeated 6 months later.

### Statistical analysis

Unless specified otherwise, all continuous data were reported as means (standard deviations). Categorical data were presented as numbers (percentages). The chi-square or Fisher’s exact test was used for categorical variables to compare two independent groups, and the independent t-test or Wilcoxon rank-sum test was used for continuous variables. For the comparison of three independent groups, one-way analysis of variance or the Kruskal–Wallis test was performed for continuous variables. Multiple comparisons were performed as a post-hoc analysis, and p-values were corrected using the Bonferroni method. McNemar’s test was used to compare changes in the proportion of patients with worsening sarcopenia between visit 1 (baseline) and visit 2 (6 months). Statistical significance was set at *p* < 0.05. All Statistical analyses were performed using R (version 4.2.1; http://cran.r-project.org/).

## Results

### Baseline characteristics

**Fig. 1:**
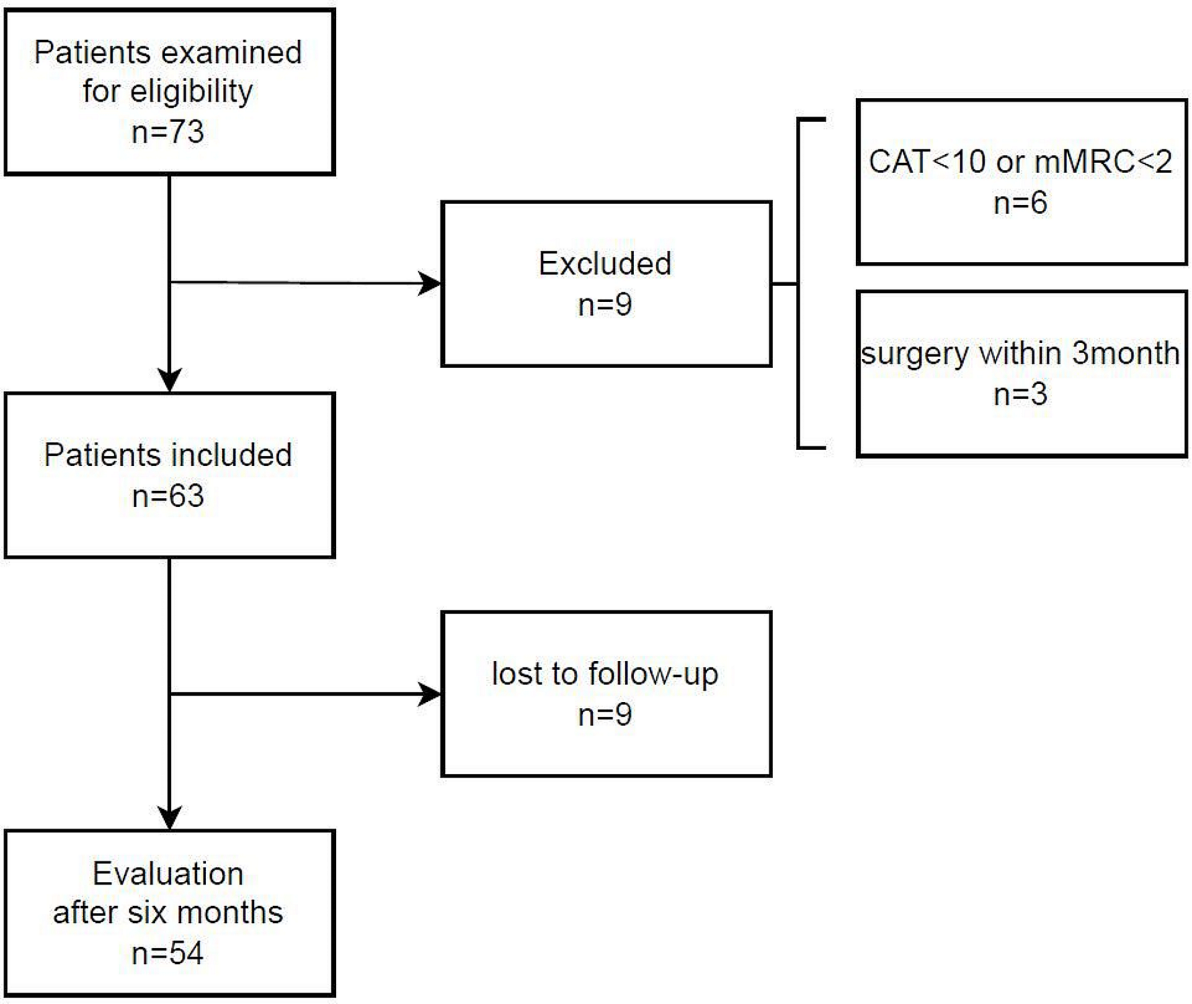
Schematic flowchart of the study

Seventy-three patients with stable COPD were recruited, and 63 eligible participants were included in this study (Fig. 1). The baseline characteristics of all the participants and subgroups are summarized in Table 1. According to the AWGS 2019 criteria, 41 (65.0%) patients were normal, 14 (22.2%) had possible sarcopenia, 6 (9.5%) had sarcopenia, and 2 (3.1%) had severe sarcopenia. The participants diagnosed with sarcopenia or severe sarcopenia were excluded from the possible sarcopenia group. The actual prevalence of sarcopenia was 12.6%, and the sarcopenia group in the analysis included patients with severe sarcopenia. The average age of the participants was 74.7 years. Most participants were ex-smokers (71.0%) with a mean smoking history of 48.4 pack years. Hypertension and cancer were the most common comorbidities. According to the GOLD guidelines, COPD stages 1 and 2 were observed in 65.1% and 30.2% of patients, respectively. CAT scores ≥ 10 were observed in 53.4% of patients. An mMRC score of 2, the indication for PR, was observed in 62.1% of patients. A score of 0–2 on the Body mass index, airflow Obstruction, Dyspnea, and Exercise (BODE) index, a scoring system for long-term outcomes of COPD, was observed in 79.4% of patients. Scores of ≤ 3 on the SARC-F, a screening tool to identify possible sarcopenia, were observed in 88.9% of patients. The percentage of participants with a history of hospitalization within the past year was 11.1%. The IPAQ was used to evaluate the amount of subjective physical activity: level 1 was defined as low, level 2 as moderate, and level 3 as high; 47.6% of patients were observed to have level 2 physical activity. Significant differences were observed in age (*p* = 0.013), body mass index (BMI, *p* = 0.014), mMRC scores (*p* = 0.026), BODE index scores (*p* = 0.035), and IPAQ scores (*p* = 0.001) between the normal and sarcopenia groups.

**Table 1:** Baseline characteristics of the study participants

Variables		Total (*n* = 63)	Normal (*n* = 41)	Possible sarcopenia (*n* = 14)	Sarcopenia (*n* = 8)	*p*-value
Age (year)		74.76 (7.19)	72.90 (7.22)	77.36 (5.76)	79.75 (5.99)	**0.013**
BMI (kg/m^2^)		23.75 (3.42)	24.11 (2.60)	24.50 (3.54)	20.54 (5.30)	**0.014**
Smoking status	Non	3 (4.8)	2 (5.0)	0 (0.0)	1 (12.5)	0.179
	Ex	44 (71.0)	27 (67.5)	13 (92.9)	4 (50.0)	
	Current	15 (24.2)	11 (27.5)	1 (7.1)	3 (37.5)	
Smoking history (pack year)		48.44 (38.68)	47.55 (40.59)	52.36 (36.24)	46.12 (36.84)	0.910
DM		9 (14.3)	7 (17.1)	2 (14.3)	0 (0.0)	0.663
HTN		21 (33.3)	16 (39.0)	4 (28.6)	1 (12.5)	0.350
CAD		13 (20.6)	10 (24.4)	2 (14.3)	1 (12.5)	0.724
BE		4 (6.3)	4 (9.8)	0 (0.0)	0 (0.0)	0.750
Tb		14 (22.2)	8 (19.5)	2 (14.3)	4 (50.0)	0.145
Cancer		21 (33.3)	15 (36.6)	4 (28.6)	2 (25.0)	0.785
GOLD	1	41 (65.1)	28 (68.3)	9 (64.3)	4 (50.0)	0.492
	2	19 (30.2)	12 (29.3)	4 (28.6)	3 (37.5)	
	3	3 (4.8)	1 (2.4)	1 (7.1)	1 (12.5)	
mMRC	0	1 (1.7)	0 (0.0)	1 (7.7)	0 (0.0)	**0.026†**
	1	8 (13.8)	7 (18.4)	0 (0.0)	1 (14.3)	
	2	36 (62.1)	25 (65.8)	8 (61.5)	3 (42.9)	
	3	9 (15.5)	6 (15.8)	2 (15.4)	1 (14.3)	
	4	4 (6.9)	0 (0.0)	2 (15.4)	2 (28.6)	
Hospitalization previous year	No	56 (88.9)	37 (90.2)	12 (85.7)	7 (87.5)	0.848
	Yes	7 (11.1)	4 (9.8)	2 (14.3)	1 (12.5)	

Continuous variables are expressed as means (standard deviations), and categorical variables are expressed as numbers (%)

One-way analysis of variance was used for continuous variables, and Fisher’s exact test (†) was used for categorical variables

BMI, body mass index; DM, diabetes mellitus; HTN, hypertension; CAD, coronary artery disease; BE, bronchiectasis; Tb, tuberculosis; GOLD, Global Initiative for Chronic Obstructive Lung Disease; mMRC, modified Medical Research Council dyspnea scale

### Clinical outcomes

Table 2 compares the results of various clinical outcomes among the groups. Diagnostic tests in the AWGS 2019 algorithm, including HGS (45.24 ± 46.11 vs. 27.95 ± 5.08, *p* < 0.001), SPPB total score (11.73 ± 0.59 vs. 10.25 ± 1.67, *p* < 0.001), and SMI (8.14 ± 0.77 vs. 6.20 ± 0.68, *p* < 0.001), showed significant differences between the normal and sarcopenia groups. Among the items of the SPPB, significant differences were observed only in gait speed (3.45 ± 0.61 vs. 3.96 ± 0.94, *p* < 0.001) and the chair stand test (9.32 ± 1.45 vs. 13.25 ± 4.44, *p* < 0.001). A significant reduction in the whole-body phase angle (WBPA) (5.51 ± 0.69 vs. 4.36 ± 0.64, *p* < 0.001) measurable using BIA was confirmed. In addition, significant decreases were observed in the sarcopenia group in the MIP (88.63 ± 22.07 vs. 53.75 ± 28.56, *p* = 0.001) and MEP (113.90 ± 33.16 vs. 74.62 ± 31.09, *p* = 0.008), related to respiratory muscle strength; PEF (363.78 ± 107.21 vs. 252.50 ± 69.02, *p* = 0.017), related to coughing ability; and 6 MWD (454.56 ± 73.30 vs. 361.88 ± 109.75, *p* < 0.001), related to exercise capacity. However, no significant differences were observed in the results of patient-reported questionnaires, such as the CAT, LCADL, SARC-F, and LINQ. On comparing the normal and possible sarcopenia groups, similar significant differences were observed in HGS, SPPB score, and 6 MWD; however, no significant differences were observed in SMI.

**Table 2:** Comparison of clinical outcomes

Variables			Total (*n* = 63)	Normal (*n* = 41)	Possible sarcopenia (*n* = 14)	Sarcopenia (*n* = 8)	*p*-value
HGS (kg)			39.39 (38.11)	45.24 (46.11)	28.79 (7.79)	27.95 (5.08)	**< 0.001†**
SPPB	Balance (second)		3.86 (0.47)	3.88 (0.40)	3.86 (0.53)	3.75 (0.71)	0.786
	GS (second)		3.69 (0.94)	3.45 (0.61)	4.23 (1.44)	3.96 (0.94)	**0.012†**
	CS (second)		10.80 (3.48)	9.32 (1.45)	13.72 (4.56)	13.25 (4.44)	< 0.001*
	Total score		11.24 (1.25)	11.73 (0.59)	10.36 (1.65)	10.25 (1.67)	< 0.001*
BIA	SMI (kg/m^2^)		7.88 (1.01)	8.14 (0.77)	8.10 (0.85)	6.20 (0.68)	< 0.001*
	Percent Body Fat		26.33 (7.81)	25.89 (6.67)	27.01 (8.43)	27.34 (12.18)	0.835
	WBPA		5.27 (0.76)	5.51 (0.69)	5.11 (0.57)	4.36 (0.64)	< 0.001*
	SMM (kg)		26.44 (3.86)	27.70 (3.20)	26.34 (2.72)	20.32 (2.71)	< 0.001*
BODE index			1.90 (1.77)	1.39 (1.14)	2.79 (2.33)	3.00 (2.39)	0.005*
CAT			11.12 (6.27)	10.13 (4.52)	11.77 (6.57)	15.29 (11.57)	0.123
SARC-F			1.51 (1.58)	1.20 (1.21)	1.86 (1.88)	2.50 (2.33)	0.065
K-LCADL			18.32 (5.62)	17.71 (4.07)	20.36 (8.03)	17.88 (7.38)	0.309
K-LINQ			11.68 (4.59)	11.37 (4.94)	11.50 (3.88)	13.62 (3.78)	0.445
K-IPAQ		Low	22 (34.9)	7 (17.1)	9 (64.3)	6 (75.0)	**0.001†**
		Moderate	30 (47.6)	25 (61.0)	4 (28.6)	1 (12.5)	
		High	11 (17.5)	9 (22.0)	1 (7.1)	1 (12.5)	
FVC%			85.81 (15.98)	86.90 (13.32)	87.36 (20.16)	77.50 (20.02)	0.293
FEV_1_%			79.22 (21.72)	79.46 (17.46)	84.71 (30.94)	68.38 (21.50)	0.238
MIP (cmH_2_O)			81.78 (25.31)	88.63 (22.07)	77.71 (21.62)	53.75 (28.56)	0.001*
MEP (cmH_2_O)			105.75 (34.61)	113.90 (33.16)	99.64 (31.01)	74.62 (31.09)	0.008*
PEF (L/min)			339.13 (107.82)	363.78 (107.21)	316.43 (102.10)	252.50 (69.02)	0.017*
6MWD (meter)			416.52 (108.72)	454.56 (73.30)	336.36 (141.54)	361.88 (109.75)	< 0.001*

Continuous variables are expressed as means (standard deviations), and categorical variables are expressed as numbers (%)

One-way analysis of variance (*) or the Kruskal–Wallis test (†) were used for continuous variables

HGS, hand grip strength; SPPB, Short Physical Performance Battery; GS, gait speed; CS, chair stand; BIA, bioelectrical impedance analysis; SMI, skeletal muscle index; WBPA, whole-body phase angle; SMM, skeletal muscle mass; BODE, Body mass index, airflow Obstruction, Dyspnea, and Exercise; CAT, chronic obstructive pulmonary disease assessment test; SARC-F, Strength, Assistance with walking, Rising from a chair, Climbing stairs, and Falling; LCADL, London Chest Activity of Daily Living; LINQ, Lung Information Needs Questionnaire; K-IPAQ, Korean version of the International Physical Activity Questionnaire; FVC, forced vital capacity; FEV_1_, forced expiratory volume in 1 s; MIP, maximum inspiratory pressure; MEP, maximum expiratory pressure; PEF, peak expiratory flow; 6 MWD, 6-minute walk distance

### Changes over 6 months

A follow-up evaluation was performed on the participants after 6 months. Nine participants were lost to follow up, and the same evaluations as those performed at baseline were performed on 54 participants (Fig. 1).

Of the nine patients who dropped out, sarcopenia was identified in one (11%) and severe sarcopenia in two (22%). After 6 months, changes in COPD severity according to the GOLD guidelines were analyzed (Table 3). After 6 months, the proportion of patients with GOLD stage 1, 2, and 3 COPD severity reduced from 64.8% (*n* = 35) to 42.6% (*n* = 23), increased from 33.3% (*n* = 18) to 48.1% (*n* = 26), and from 1.9% (*n* = 1) to 9.3% (*n* = 5), respectively, with statistically significant differences observed between the initial and follow-up test. The severity of sarcopenia was analyzed according to the AWGS 2019 criteria (Table 3). After 6 months, the proportion of patients in the normal group changed from 66.7% (*n* = 36) to 75.9% (*n* = 41), while that of those with possible sarcopenia changed from 24.1% (*n* = 13) to 20.4% (*n* = 11), and that of those with sarcopenia from 9.3% (*n* = 5) to 3.7% (*n* = 2); however, the differences were not statistically significant. Patients with severe sarcopenia were included in the sarcopenia group because of the small sample size.

**Table 3:** Changes in COPD and sarcopenia severity after 6 months assessed using McNemar’s test

(a)						
		Visit 2 (6 month)				
Visit 1 (baseline)		GOLD 1	GOLD 2	GOLD 3	Visit 1 total	*p*-value
GOLD stage	GOLD 1	23 (42.6)	11 (20.4)	1 (1.9)	35 (64.8)	0.003
	GOLD 2	0 (0.0)	14 (25.9)	4 (7.4)	18 (33.3)	
	GOLD 3	0 (0.0)	1 (1.9)	0 (0.0)	1 (1.9)	
	Visit 2 total	23 (42.6)	26 (48.1)	5 (9.3)	54 (100.0)	

(b)						
		Visit 2 (6 month)				
Visit 1 (baseline)		Normal	Possible sarcopenia	Sarcopenia	Visit 1 total	*p*-value
AWGS 2019	Normal	33 (61.1)	3 (5.6)	0 (0.0)	36 (66.7)	0.261
	Possible sarcopenia	6 (11.1)	7 (13.0)	0 (0.0)	13 (24.1)	
	Sarcopenia	2 (3.7)	1 (1.9)	2 (3.7)	5 (9.3)	
	Visit 2 total	41 (75.9)	11 (20.4)	2 (3.7)	54 (100.0)	

*n* = 54

Categorical variables are expressed as numbers (%)

GOLD, Global Initiative for Chronic Obstructive Lung Disease; AWGS, Asian Working Group for Sarcopenia

## Discussion

To the best of our knowledge, this is the first study to use the AWGS 2019 criteria in older adult patients with stable COPD in Korea. The prevalence of sarcopenia in this study was 12.6%, lower than the 14.5% reported in a previous study from the UK and 25% reported in a study from Korea [9, 12]. These results were obtained because the EWGSOP criteria used in the two previous studies were based on lower BIA and HGS cutoff values than the AWGS 2019 criteria. In addition, differences in diagnostic algorithms may have influenced the changes in prevalence. In a previous study using the AWGS 2019 criteria, the prevalence of sarcopenia in males aged over 70 years was reported to be 21.3%, which is higher than that in this study [8]. However, it should be noted that the prevalence of sarcopenia varies significantly depending on the method of applying the various assessments presented in the algorithm. Low muscle mass is a prerequisite for diagnosing sarcopenia according to the AWGS 2019 criteria, and only one of the two criteria, that for muscle strength or that for physical performance, must be met. Physical performance can be evaluated via any of the following: gait speed, 5TSTS test, and the SPPB. A previous study reported that the prevalence of sarcopenia was 14.4% based on HGS, 13.3% based on gait speed, 15.6% based on the 5TSTS test, 6.6% based on the SPPB, and 26.8% when all evaluations were reflected [8]. In this study, among the eight patients diagnosed with sarcopenia, two had an HGS < 28 kg, two had a gait speed < 1.0 m/s, four had a 5TSTS test time of ≥ 12s, and one had an SPPB score ≤ 9. This means that because differences in prevalence may occur due to the limitations of the algorithm, a detailed review of which criterion was used and which evaluation tool was used for the same criterion is required to interpret or compare the prevalence. When calculating the prevalence of sarcopenia (12.6%), defined as the fulfillment of the HGS or physical performance criteria, participants diagnosed with severe sarcopenia (3.1%) were included. In conclusion, the prevalence rate of sarcopenia in this study (12.6%) is lower than that of older adults aged ≥ 70 years in the previous Korean study (26.8%) [8]. This might be because patients who visited the respiratory center regularly and received medical treatment were the main participants. In addition, we could not rule out a selection bias because of the participants’ relatively high adherence to medical treatment and ability to perform the 6 MWT.

In the AWGS 2019 algorithm, three screening tools–calf circumference, SARC-F, and SARC-Calf–are used to diagnose possible sarcopenia in primary healthcare settings. Possible sarcopenia is defined as low muscle strength or reduced physical performance. In this study, SARC-F evaluation was unnecessary because this was a clinical research setting, and the participants had already been diagnosed with COPD; however, the SARC-F was administered to obtain additional information. The SARC-F consists of five items: strength, assistance with walking, rising from a chair, climbing stairs, and falls [16]. The Korean version of the SARC-F has also been verified, and its association with adverse clinical outcomes has been reported [22, 23]. In the algorithm, a SARC-F score of ≥ 4 was used as the criterion for diagnosing sarcopenia, and additional tests are required in these patients because of the low sensitivity and high specificity of the SARC-F [24]. In this study, when the SARC-F was administered to all participants, only seven participants had a SARC-F score of ≥ 4. This result indicates the limited role of the SARC-F as a community screening tool. Therefore, this study diagnosed possible sarcopenia using the HGS or the 5TSTS test, regardless of the SARC-F score. Based on this criterion, possible sarcopenia was present in 34.9% and 22.2% of the patients using the HGS and 5TSTS test respectively, excluding those diagnosed with sarcopenia. In this study, 22.2% of the participants were suspected of having sarcopenia but were not diagnosed and were analyzed as one group to identify their characteristics.

When baseline characteristics of the participants were compared among groups (Table 1), significant differences in age and BMI were observed, as in a previous study [25, 26]. Consequently, it can be expected that old age and low BMI at the first visit strongly predict sarcopenia. There were no significant differences in the underlying diseases, smoking status, or amount of smoking. In the evaluation of the patient’s subjective symptoms, no significant difference was observed in CAT scores; however, mMRC scores were significantly higher in the sarcopenia group than in the normal group, similar to the results of previous studies [12]. The higher the mMRC scores, the lower the activity level due to severe dyspnea, resulting in secondary muscle loss [27]. Because the CAT included dyspnea, inactivity, and other factors, such as cough, sputum, and sleep, it was concluded that sarcopenia alone did not cause a significant difference in CAT scores. In patients with COPD, a higher BODE index suggests a worse prognosis [28]. Since the 6 MWD, BMI, mMRC score, and FEV_1_ are included in this index, it is strongly correlated with the patient’s exercise capacity, and a significant difference in the rate was observed between the normal and sarcopenia groups [29, 30]. The IPAQ, a verified questionnaire for evaluating physical activity in older adults, has been translated into Korean [31]. Patients can be classified into three IPAQ activity levels (low, moderate, and high) based on its method of calculation; in this study, the number of patients with low activity levels was significantly higher in the sarcopenia group than the normal group (17.1% vs. 75%). These results indicate that close follow-up for sarcopenia is recommended for patients with low IPAQ activity levels.

Table 2 shows the differences among the groups in the findings for various evaluation tools. For HGS, a significant difference was observed. The result of the 5TSTS test, a physical performance test, showed a significant difference between the normal and possible sarcopenia and the normal and sarcopenia groups. However, a difference in gait speed was observed only between the normal and possible sarcopenia groups. The SPPB total score showed significant differences among the three groups, and the average value in the sarcopenia group was 10.25 ± 1.67, exceeding the cutoff value in the AWGS 2019 criteria. In our study, the 5TSTS test diagnosed sarcopenia at the highest rate. Therefore, among the various physical performance evaluation tools, we recommend that the 5TSTS test be preferred in primary care settings, as it is easy to perform and requires little space. Among the BIA parameters, SMI and SMM showed significant differences between the normal and sarcopenia groups. The differences between these variables matched the design of the AWGS 2019 algorithm. Notably, among the other BIA variables, the WBPA significantly decreased in the sarcopenia group (5.51 vs. 4.36). The WBPA is calculated as the ratio of reactance to resistance and has been highlighted as a relative indicator of cellular mass, membrane integrity, and hydration status [32, 33]. A low WBPA has been identified as a predictor of mortality in patients with various clinical conditions, including lung disease [34]. The phase angle can be used to predict sarcopenia; however, the cutoff value remains to be established [35]. In patients with COPD, a higher association was observed between the WBPA and FEV_1_ than between the SMI and FEV_1_ in a linear regression model [36]. This indicates that the WBPA in patients with COPD has not yet been established as a diagnostic criterion for sarcopenia; however, it has sufficient value as a clinical index to be observed together with SMI.

Table 2 shows that significant differences were observed in MIP, MEP, PEF, and 6 MWD. MIP and MEP are indicators of inspiratory and expiratory muscle strength, respectively. In patients with COPD, various clinical studies have confirmed significant effects of respiratory muscle training on exercise capacity and symptoms [37–39]. A study on the association between MIP, MEP, and sarcopenia reported that sarcopenia could be diagnosed using MIP ≤ 55 cmH_2_O and MEP ≤ 60 cmH_2_O as cutoff points in males [40]. Additionally, an attempt was made recently to define the term *respiratory sarcopenia* as a weakness of respiratory muscles in patients with existing sarcopenia [41]. In patients diagnosed with whole-body sarcopenia according to the existing AWGS 2019 criteria, respiratory sarcopenia can be diagnosed if there is low respiratory muscle strength and/or low respiratory function and related underlying diseases. *Low respiratory muscle strength* is defined as an MIP ≤ 80 cmH_2_O [41]. In this study, the MIP was significantly lower (88.63 vs. 53.75 cmH_2_O) in the sarcopenia group than in the normal group, confirming its value as an additional diagnostic tool for sarcopenia and respiratory sarcopenia. The PEF has also been proposed as an evaluation tool for respiratory sarcopenia. However, it may be underestimated in patients with obstructive diseases, such as COPD, making it unsuitable as a diagnostic tool for older adults [41, 42]. However, in this study, which only included patients with COPD, significant decreases were observed in PEF and MEP. Therefore, further research is needed on PEF and MEP value as a diagnostic tool for sarcopenia.

The 6 MWT is a well-known tool for assessing the prognosis of COPD, for determining the response to medication or rehabilitation, and for use as a submaximal test for exercise capacity [43]. Limited mobility in patients with sarcopenia is defined as a 6 MWD < 400 m [44]. In this study, there were no significant differences in the GOLD stage or FEV_1_ among the groups. However, the result of the 6 MWD was lower (454.56 vs. 361.88 m) in the sarcopenia group than the normal group; therefore, it can be used as an indirect diagnostic indicator of sarcopenia. In other patient-reported questionnaires, no significant differences were observed among the groups because many items other than sarcopenia were included.

Table 3 presents the changes in the GOLD stage and COPD severity when the participants were re-evaluated 6 months later. Nine patients dropped out, and 54 were re-evaluated. Interestingly, the GOLD stage, stratified based on the FEV_1_, showed significant deterioration after 6 months. Previous studies have reported that in patients with COPD with GOLD stages 2 and 3, the FEV_1_ naturally decreased by 47–79 ml/year and 56–59 ml/year, respectively [45]. None of the patients who were followed up had a history of hospitalization in the 6-month follow-up period. However, owing to the characteristics of patients visiting a tertiary respiratory hospital, we considered that they had many comorbidities, and acute exacerbations were relatively common. Additionally, when comparing the mean FEV_1_ values between the two visits, no significant differences were observed, which could be interpreted as an effect of the classification system used (see Additional file 1). No statistically significant change was found in sarcopenia severity during the 6-month follow-up period. Although the difference in the proportion of sarcopenia was not statistically significant, the overall proportion appeared to have slightly improved. Because it was not an intervention study, it is difficult to clearly know the factors that affect the improvement in sarcopenia rates. However, most participants who received appropriate medical treatment had relatively high activity levels. In addition, the nine dropouts included two patients with severe sarcopenia and one with sarcopenia; therefore, the 54 patients who were re-evaluated were expected to be relatively healthy. From this result, it can be interpreted that the prevalence of sarcopenia did not increase significantly during the 6-month period in older adult patients with stable COPD who regularly visited outpatient clinics. Changes over 6 months were compared for various parameters, and gait speed (3.69 vs. 4.08, *p* < 0.001) was the only parameter that showed statistically significant deterioration (see Additional file 1).

PR in patients with COPD has been reported to have various effects, such as improved quality of life, dyspnea, and exercise capacity [46]. According to the British Thoracic Society guidelines, outpatient respiratory rehabilitation treatment is recommended if the mMRC score is ≥ 2 [47]. In patients with sarcopenia, exercise has been proven effective in improving exercise capacity, muscle strength, and muscle mass [7]. Therefore, sarcopenia and COPD have overlapping clinical characteristics and rehabilitation strategies, and if appropriate evaluation and PR are established, simultaneous improvement will be possible.

This study had some limitations. First, the short follow-up period may not have been sufficient to observe the natural course of sarcopenia. In previous studies, an improvement in the prevalence of sarcopenia was observed when the period immediately after COPD exacerbation and that after 6 months were compared, and the prevalence of sarcopenia increased in older adults after 4 years of follow-up [48, 49]. Since this study only included a follow-up period of 6 months in patients with stable COPD, it is considered that the period was not sufficient to examine such a difference. Second, selection bias could not be ruled out because the participants were recruited from a tertiary hospital. Therefore, the results of this study are limited to representing the general characteristics of patients with COPD. Patients who visit tertiary hospitals often present with various comorbidities that affect sarcopenia. However, they also have relatively high adherence to medicine; that is, they are quite interested in their health. Lastly, it is difficult to explain the significant improvement in PEF after 6 months in all participants. Although we did not observe a factor such as timing of inhaler use, it is possible that it may has influenced the result.

## Conclusions

A relatively lower prevalence of sarcopenia (12.6%) was observed in this study than in a previous study involving older adult outpatients with stable COPD. Patients with sarcopenia tended to be older, had a higher BODE index and mMRC score and lower BMI and activity levels than the patients without sarcopenia. The WBPA, MIP, MEP, PEF, and 6 MWD are additional diagnostic parameters for sarcopenia. In patients with stable COPD, no significant change in the prevalence of sarcopenia was observed during the 6-month follow-up period. These results provide valuable information for establishing outpatient PR strategies in Korea.

### Electronic supplementary material

Below is the link to the electronic supplementary material.

Supplementary Material 1

## Data Availability

The datasets used and analyzed in this study are available from the corresponding author upon reasonable request.

## Abbreviations

5TSTS: 5-time sit-to-stand
6 MWT: 6-minute walk test
6 MWD: 6-minute walk distance
AWGS: Asian Working Group for Sarcopenia
BIA: bioelectrical impedance analysis
BODE: Body mass index, airflow Obstruction, Dyspnea, and Exercise
CAT: chronic obstructive pulmonary disease assessment test
COPD: chronic obstructive pulmonary disease
EWGSOP: European Working Group on Sarcopenia in Older People
FVC: forced vital capacity
FEV_1_: forced expiratory volume in 1 s
GOLD: Global Initiative for Chronic Obstructive Lung Disease
HGS: hand grip strength
IPAQ: International Physical Activity Questionnaire
IRB: Institutional Review Board
LCADL: London Chest Activity of Daily Living
LINQ: Lung Information Needs Questionnaire
MEP: maximal expiratory pressure
MIP: maximal inspiratory pressure
mMRC: modified Medical Research Council
PEF: peak expiratory flow
PR: pulmonary rehabilitation
SMI: skeletal muscle mass index
SMM: skeletal muscle mass
WBPA: whole-body phase angle
