# Supplementary material for: Prevalence and clinical characteristics of Sarcopenia in older adult patients with stable chronic obstructive pulmonary disease: a cross-sectional and follow-up study
Source: BMC Pulm Med. 2024 May 2;24:219. doi: 10.1186/s12890-024-03034-5 (PMC11067242; doi:10.1186/s12890-024-03034-5)
Supplement: Supplementary file 1 — Supplementary Material 1 [file 12890_2024_3034_MOESM1_ESM.docx]

Additional file 1. Differences in baseline and follow-up clinical outcomes

| Variables |  | n | Visit 1  (baseline) | Visit 2 (6month) | Difference  (95%CI) | p-value |
| --- | --- | --- | --- | --- | --- | --- |
| HGS (kg) | | 54 | 40.53 (40.98) | 42.95 (55.17) | 2.42 (-15.867, 20.704) | 0.792 |
| SPPB | Balance (second) | 54 | 3.87 (0.44) | 3.94 (0.23) | 0.07 (-0.043, 0.191) | 0.209 |
|  | GS (second) | 54 | 3.69 (0.97) | 4.08 (1.14) | 0.39 (0.194, 0.582) | **<0.001** |
|  | CS (second) | 54 | 10.73 (3.30) | 10.05 (2.86) | -0.68 (-1.333, -0.037) | **0.039** |
|  | Total score | 54 | 11.28 (1.16) | 11.33 (1.47) | 0.06 (-0.172, 0.283) | 0.626 |
| BIA | SMI (kg/m^2^) | 53 | 8.01 (0.98) | 8.08 (1.00) | 0.06 (-0.215, 0.343) | 0.646 |
|  | Percent Body Fat | 53 | 25.69 (7.44) | 24.38 (7.63) | -1.31 (-3.013, 0.406) | 0.132 |
|  | WBPA | 53 | 5.29 (0.74) | 4.46 (5.85) | -0.84 (-2.399, 0.727) | 0.288 |
|  | SMM (kg) | 53 | 26.91 (3.74) | 27.14 (3.81) | 0.23 (-0.547, 1.011) | 0.553 |
| BODE index | | 54 | 1.78 (1.55) | 1.39 (1.39) | -0.39 (-0.722, -0.056) | **0.023** |
| CAT | | 54 | 10.54 (5.05) | 11.33 (5.81) | 0.79 (-0.715, 2.292) | 0.297 |
| SARC-F | | 54 | 1.33 (1.33) | 1.78 (1.44) | 0.44 (0.176, 0.713) | **0.002** |
| K-LCADL | | 54 | 18.22 (5.47) | 19.57 (6.34) | 1.35 (0.201, 2.503) | **0.022** |
| K-LINQ | | 54 | 11.41 (4.44) | 9.35 (4.80) | -2.06 (-2.962, -1.149) | **<0.001** |
| FVC% | | 54 | 86.94 (15.01) | 85.81 (13.80) | -1.13 (-3.096, 0.837) | 0.254 |
| FEV_1_% | | 54 | 79.94 (20.85) | 78.65 (20.17) | -1.3 (-4.271, 1.678) | 0.386 |
| MIP (cmH_2_O) | | 52 | 83.79 (22.44) | 86.04 (25.87) | 2.25 (-1.088, 5.588) | 0.182 |
| MEP (cmH_2_O) | | 52 | 105.17 (33.37) | 106.38 (40.71) | 1.21 (-5.461, 7.884) | 0.717 |
| PEF (L/min) | | 54 | 342.50 (106.17) | 363.70 (134.41) | 21.2 (1.993, 40.414) | **0.031** |
| 6MWT (meter) | | 53 | 423.02 (104.25) | 436.25 (91.01) | 13.23 (-0.912, 27.365) | 0.066 |
| Continuous variables are expressed as means (standard deviations)  The paired t-test was used for continuous variables  HGS, hand grip strength; SPPB, Short Physical Performance Battery; GS, gait speed; CS, chair stand; BIA, bioelectrical impedance analysis; SMI, skeletal muscle index; WBPA, whole-body phase angle; SMM, skeletal muscle mass; BODE, Body mass index, airflow Obstruction, Dyspnea, and Exercise; CAT, chronic obstructive pulmonary disease assessment test; SARC-F, Strength, Assistance with walking, Rising from a chair, Climbing stairs, and Falling; LCADL, London Chest Activity of Daily Living; LINQ, Lung Information Needs Questionnaire; FVC, forced vital capacity; FEV_1_, forced expiratory volume in 1 s; MIP, maximum inspiratory pressure; MEP, maximum expiratory pressure; PEF, peak expiratory flow; 6 MWT, 6-minute walk test | | | | | | |

Additional file 2. Differences in baseline and follow-up clinical outcomes between groups

| Variables |  | Normal  (n=36) | Sarcopenia  (n=18) | p-value^a^ | p-value^b^ | |
| --- | --- | --- | --- | --- | --- | --- |
| HGS (kg) | | 3.01 (82.41) | 1.24 (3.11) | 0.898 | 0.219 | |
| SPPB | Balance (second) | 0.08 (0.37) | 0.06 (0.54) | 0.846 | 0.460 | |
|  | GS (second) | 0.38 (0.81) | 0.41 (0.49) | 0.835 | 0.463 | |
|  | CS (second) | -0.15 (2.11) | -1.76 (2.56) | **0.029** | **0.013** | |
|  | Total score | -0.08 (0.87) | 0.33 (0.69) | 0.062 | 0.057 | |
| BIA | SMI (kg/m^2^) | -0.16 (0.96) | 0.49 (0.99) | **0.028** | **0.020** | |
|  | Percent Body Fat | 0.91 (4.51) | -5.61 (6.88) | **0.001** | **<0.001** | |
|  | WBPA | -0.12 (0.41) | -2.22 (9.74) | 0.374 | 0.381 | |
|  | SMM (kg) | -0.53 (2.04) | 1.71 (3.55) | **0.021** | **0.013** | |
| BODE index | | -0.39 (1.05) | -0.39 (1.54) | 1.000 | 0.670 | |
| CAT | | 0.34 (5.14) | 1.71 (5.96) | 0.426 | 0.930 | |
| SARC-F | | 0.33 (0.96) | 0.67 (1.03) | 0.259 | 0.234 | |
| K-LCADL | | 0.89 (4.11) | 2.28 (4.39) | 0.271 | 0.347 | |
| K-LINQ | | -2.11 (3.25) | -1.94 (3.56) | 0.868 | 0.978 | |
| FVC% | | 0.75 (6.03) | -4.89 (8.04) | **0.014** | **0.009** | |
| FEV_1_% | | 1.56 (7.28) | -7.00 (14.48) | **0.028** | **0.020** | |
| MIP (cmH_2_O) | | 0.85 (11.21) | 4.89 (13.27) | 0.280 | 0.603 | |
| MEP (cmH_2_O) | | 2.21 (24.53) | -0.67 (23.44) | 0.682 | 0.281 | |
| PEF (L/min) | | 23.19 (69.11) | 17.22 (74.74) | 0.778 | 0.581 | |
| 6MWT (meter) | | 4.69 (30.29) | 29.83 (75.97) | 0.192 | 0.367 | |
| Continuous variables are expressed as means (standard deviations)  a: Independent T-test; b: Wilcoxon rank sum test  HGS, hand grip strength; SPPB, Short Physical Performance Battery; GS, gait speed; CS, chair stand; BIA, bioelectrical impedance analysis; SMI, skeletal muscle index; WBPA, whole-body phase angle; SMM, skeletal muscle mass; BODE, Body mass index, airflow Obstruction, Dyspnea, and Exercise; CAT, chronic obstructive pulmonary disease assessment test; SARC-F, Strength, Assistance with walking, Rising from a chair, Climbing stairs, and Falling; LCADL, London Chest Activity of Daily Living; LINQ, Lung Information Needs Questionnaire; FVC, forced vital capacity; FEV_1_, forced expiratory volume in 1 s; MIP, maximum inspiratory pressure; MEP, maximum expiratory pressure; PEF, peak expiratory flow; 6 MWT, 6-minute walk test | | | | | |  |
